# Synthesis of Fluorous Ferrofluids and Effects of the Nanoparticle Coatings on Field- and Temperature-Dependent Magnetizations

**DOI:** 10.1021/acs.chemmater.3c01172

**Published:** 2023-09-29

**Authors:** Fang-Chu Lin, Heidi L. van de Wouw, Otger Campàs, Ellen M. Sletten, Jeffrey I. Zink

**Affiliations:** † Department of Chemistry and Biochemistry, University of California Los Angeles, Los Angeles, California 90095, United States; ‡ California Nanosystems Institute, University of California Los Angeles, Los Angeles, California 90095, United States; § Cluster of Excellence Physics of Life, TU Dresden, Dresden 01307, Germany; ∥ Max Planck Institute of Molecular Cell Biology and Genetics, Dresden 01307, Germany; ⊥ Center for Systems Biology Dresden, Dresden 01307, Germany; # Department of Mechanical Engineering, University of California Santa Barbara, Santa Barbara, California 93106, United States

## Abstract

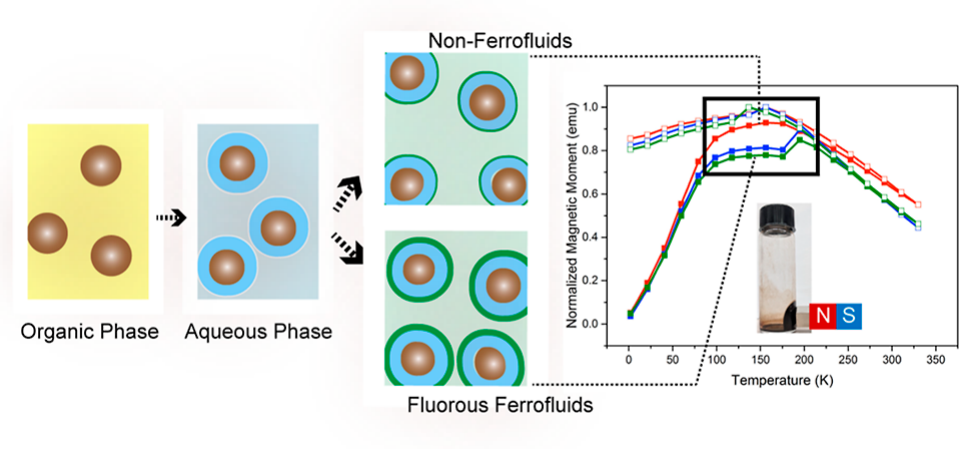

Ferrofluids have been extensively employed in industrial, environmental, and biomedical areas. Among them, fluorous ferrofluids are of particular interest because of the biorthogonal nature of perfluorocarbons (PFCs). However, the noninteracting nature of PFCs as well as challenges in functionalization of nanoparticle surfaces with fluorous ligands has limited their applications, especially in biomedicine. In particular, commercially available fluorous ferrofluids are stabilized using ionic surfactants with charged groups that physically interact with a wide range of charged biological molecules. In this paper, we developed a unique two-phase ligand attachment strategy to render stable fluorous ferrofluids using nonionic surfactants. The superparamagnetic Fe_3_O_4_ or MnFe_2_O_4_ core of the magnetic nanoparticles, the magnetic component of the ferrofluid, was coated with a silica shell containing abundant surface hydroxyl groups, thereby enabling the installation of fluorous ligands through stable covalent, neutral, siloxane bonds. We explored chemistry–material relationships between different ligands and PFC solvents and found that low-molecular-weight ligands can assist with the installation of high-molecular-weight ligands (4000–8000 g/mol), allowing us to systematically control the size and thickness of ligand functionalization on the nanoparticle surface. By zero-field-cooled magnetization measurements, we studied how the ligands affect magnetic dipole orientation forces and observed a curve flattening that is only associated with the ferrofluids. This work provided insight into ferrofluids’ dependence on interparticle interactions and contributed a methodology to synthesize fluorous ferrofluids with nonionic surfactants that exhibit both magnetic and chemical stability. We believe that the doped MnFe_2_O_4_ fluorous ferrofluid has the highest combination of stability and magnetization reported to date.

## Introduction

Ferrofluids are extremely versatile nanomaterials possessing both magnetic properties and liquid behavior. First developed by NASA in the 1960s, they consist of single-domain magnetic nanoparticles (NPs) in a carrier liquid. The unique properties of ferrofluids have led to their use in a wide range of applications, including in hard drives, speakers, and sensors. More recently, ferrofluids have seen use in the biomedical applications (including drug delivery, hyperthermia cancer therapy, imaging, and biosensing), engineering (including sealing, heat transfer fluids, lubrication, and film bearing, digital microfluids), and environmental fields (for wastewater and fugitive emission treatments).^1–5^ In addition to the compositional, structural, and magnetic characteristics of magnetic NPs, the properties of ferrofluids strictly depend on the colloidal stability of the NPs in the carrier liquid.^6^ The liquid media of interest in this work are bio-orthogonal perfluorocarbon (PFC) oils. Because of their unique properties such as their repellency of both organic- and aqueous-based solutions, PFCs are being used in medical applications such as immobilized liquid layers,^7^ in clinical applications such as vitreoretinal tamponade,^8^ and as blood substitutes.^9^ PFC-based nanoemulsions have also been utilized as therapeutic agents to carry photosensitizers^10^ and deliver genes^11^ and as diagnostic agents for ^19^F-MRI^12–14^ and ultrasound (US)^15,16^ imaging.^17^ With combined properties of both PFCs and ferrofluids, PFC-based fluorous ferrofluids have enormous potential in biomedical applications. For example, fluorous ferrofluids have been utilized as constriction elements to help control intraocular pressure,^18^ as acoustic wave resonators^16^ that can be potentially used in US imaging, as antimicrobial/antifungal agents, and also for drug delivery and MRI imaging. In addition, the PFC oil microdroplets containing fluorous ferrofluids are used as force sensors as well as mechanical actuators and microrheometers within living tissues, enabling direct measurements of cell-generated mechanical stresses and the local tissue material properties of the cellular microenvironment within developing tissues.^19^ Despite their successful applications in biomedicine, very few fluorous ferrofluids exist largely due to the difficulty in systematic control of the ligands to ensure the stability of NPs in liquid media.

The stability of ferrofluids depends highly on the ligands attached to the NPs, which have two major functions: (1) introduce a distance and steric repulsion between the magnetic NPs to overcome the forces of attraction caused by van der Waals forces and magnetic attraction, preventing agglomeration of magnetic NPs, and (2) serve as the outer layer of the magnetic NPs that is compatible with the liquid carrier, rendering the NPs soluble in such liquid carrier.^20,21^ The conventional strategies to synthesize fluorinated magnetic NPs have been based on the exchange of the ligands on the magnetic NP surface with fluorinated ones.^22,23^ Using this ligand-exchange approach, the fluorinated ligands were reversibly bound to the NPs, and their leaching could be disruptive to the surrounding environment. This is particularly concerning for *in vivo* experiments. To expand the applications of fluorous ferrofluids, especially in biomedical applications, it is essential to control the chemical functionalization of the magnetic NPs with appropriate surface chemistry.

Many biomedical applications require ferrofluids to have well-controlled surface chemistry to ensure the proper interactions with biological agents, such as molecules, cells, and tissues. The bioorthogonal nature of PFCs is perfectly suited for applications in biomedicine, and PFC oils have been extensively used for this reason. However, commercially available PFC ferrofluids contain ionic surfactants to ensure their stability. The existence of charged groups in the ferrofluid can lead to undesired interactions with many charged molecules in cells and tissues, either altering the biological system or precluding the desired use of ferrofluid. It remains a challenge to develop PFC ferrofluids with nonionic surfactants that would provide an optimal inert chemical environment for biological applications.

In this paper, we introduce new nonionic fluorous ferrofluids with fluorous ligands chemically bonded to silica coatings on superparamagnetic NP surfaces through stable covalent chemistry. To prepare the fluorous ferrofluids, we build on seminal biphasic catalysis methods first reported by Horváth in 1994.^24^ In these works, catalysts were sequestered in the fluorous phase through the use of fluorous tags, which enabled facile purification of products from the catalyst.^25^ As these methods developed, an array of fluorous tags with spacers that did not affect the overall reactivity were introduced.^25,26^ Here, we use a biphasic fluorous/organic mixture to attach fluorous ligands with molecular weights (MWs) that differ by more than a factor of 10 and to systematically control the surface ligands conjugated on NPs. We demonstrate that larger sized fluorous ligands with a MW of 4000–8000 g/mol can be grafted on the NP surface with the assistance of low-MW fluorous ligands. The lengths of the ligands used are different, allowing us to probe the influence of the interparticle distance on the magnetic dipole forces and to study the interplay between NPs and the carrier liquid for the optimization of ferrofluid synthesis. The biphasic synthesis also enables facile purification of the NPs because once sufficient ligand exchange has occurred, the NPs associate only with the fluorous phase.

## Results and Discussion

### Overview of the Synthetic Strategy

**Figure 1:**
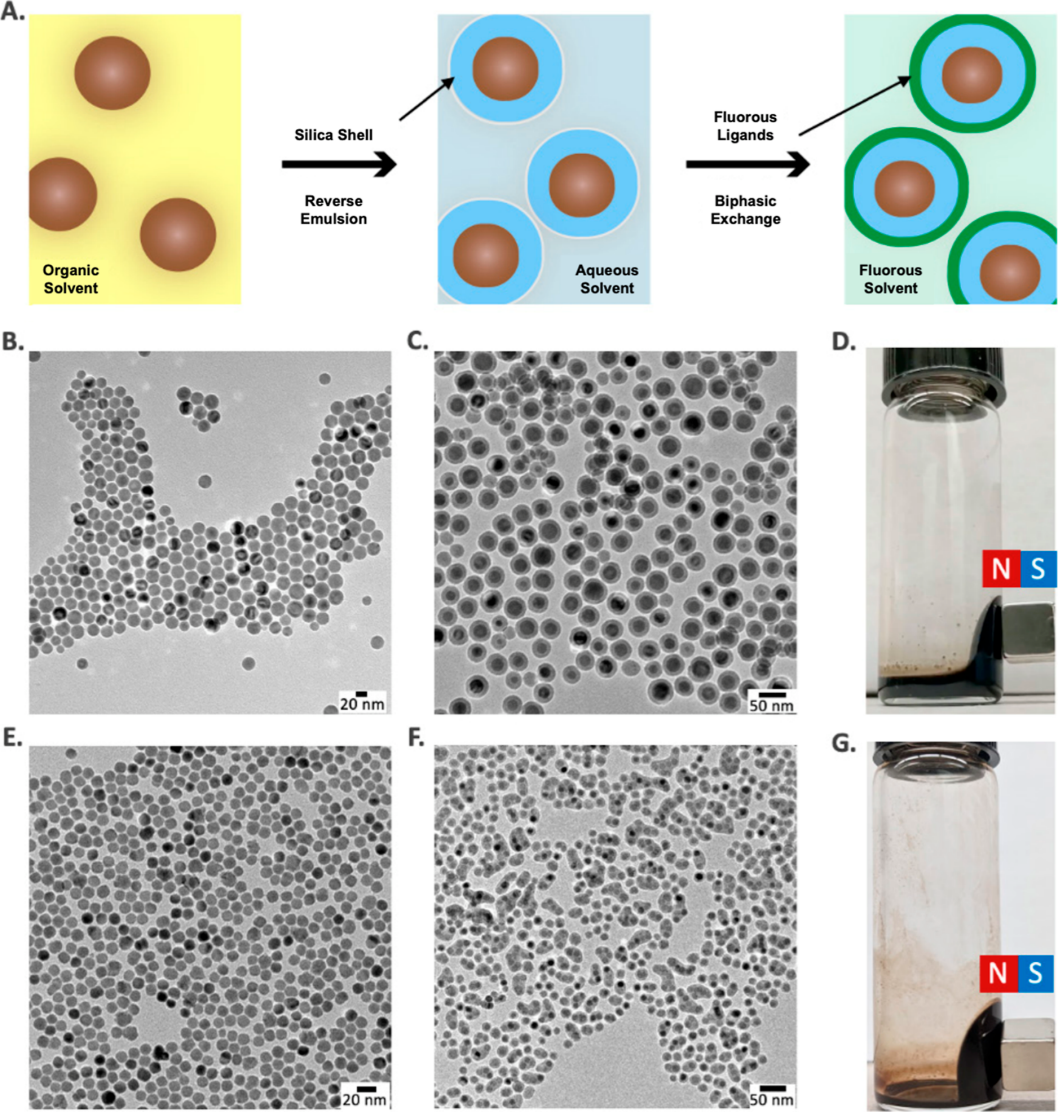
(A) Schematic illustration of fluorinated ferrofluids synthesis by first coating hydrocarbon-oil-based magnetic core (Fe_3_O_4_ NPs or MnFe_2_O_4_ NPs) with a thin layer of silica, forming the so-called core–shell NPs (Fe_3_O_4_@SiO_2_ NPs or MnFe_2_O_4_@SiO_2_ NPs). The abundant hydroxyl (−OH) groups allow the installation of fluorous ligands onto the NP surface, resulting in fluorous ferrofluids. TEM image of (B) Fe_3_O_4_ NPs, (C) Fe_3_O_4_@SiO_2_ NPs, (E) MnFe_2_O_4_ NPs, and (F) MnFe_2_O_4_@SiO_2_ NPs. Photos of fluorous ferrofluids’ response to a neodymium magnet using (D) Fe_3_O_4_ NPs or (G) MnFe_2_O_4_ NPs as the magnetic core. Organic solvent = hexane; polar solvent = ethanol; and fluorous solvent = HFE-7700.

The synthetic steps of nonionic fluorous ferrofluids’ preparation are shown in Figure 1A, with full details described in the Experimental Section. First, monodisperse Fe_3_O_4_ NPs (Figure 1B), the magnetic component of the ferrofluids, were synthesized by a modified thermal decomposition method.^27,28^ Fe_3_O_4_ NPs were stabilized by oleic acid and, therefore, can be suspended in nonpolar hydrocarbon solvents such as hexane. The Fe_3_O_4_ core was coated with a SiO_2_ shell forming core–shell Fe_3_O_4_@SiO_2_ NPs, where the abundant presence of surface hydroxyl (−OH) groups on the SiO_2_ shell enabled the attachment of fluorous ligands via stable covalent siloxane chemistry. Growth of a uniform silica shell onto the Fe_3_O_4_ core was accomplished using a reverse microemulsion method.^29^ Transmission electron microscopy (TEM) was performed for direct observation of the core–shell NPs consisting of an iron oxide core and the surrounding silica shell (Figure 1C). The average sizes of Fe_3_O_4_@SiO_2_ NPs were determined: diameter of the Fe_3_O_4_@SiO_2_ NPs (42.5 ± 2.6 nm) by TEM, Figure 1C; and 60 nm by dynamic light scattering (DLS, Figure S1), the diameter of the iron oxide core (27.2 ± 3.8 nm by TEM), and the thickness of the silica shell (7.5 ± 2.1 nm). The TEM image also showed that the majority of monodisperse Fe_3_O_4_@SiO_2_ NPs were single core. The thickness of the silica shell is tunable and was chosen to coat the Fe_3_O_4_ core to maximize the magnetic ratio of the core/shell NPs, i.e., to generate higher magnetization overall. By tuning the amount of TEOS and NH_4_OH, the SiO_2_ shell thickness was varied from >10 nm (Figure S2A) to <5 nm (Figures 1C and S2).^29^ It is worth noting that if only the amount of TEOS was tuned, a rough NP surface would be attained (Figure S2B,C). To grow a thin shell with even and full coverage of the Fe_3_O_4_ core, the water phase generated by NH_4_OH was adjusted carefully to provide a more confined space for the silica shell-forming process (Figure S2D–F). The Fe_3_O_4_@SiO_2_ NPs can be dispersed in polar solvents such as ethanol, and their surfaces can be functionalized with fluorous ligands via Si–O–Si bonds. The resulting magnetic NPs were compatible with the PFC liquid carrier, forming the fluorous ferrofluids shown in Figure 1D.

The synthetic methods developed for making fluorous ferrofluids using the familiar superparamagnetic Fe_3_O_4_ cores in a silica shell carry over to other superparamagnetic cores. For many applications, stronger magnetic field responses are desirable. To demonstrate the transferability of the coating methodology and carry out an investigation of the temperature-dependent magnetization (M–T) properties, we doubled the magnetic moment of the NPs by synthesizing manganese-doped iron oxide NPs (MnFe_2_O_4_ NPs) and used the same silica-coating methodology. The MnFe_2_O_4_ NPs (15.4 ± 1.2 nm by TEM, Figure 1E) were obtained by synthesizing 8.4 nm MnFe_2_O_4_ NPs (the seeds, Figure S9A) followed by a second deposition of MnFe_2_O_4_.^30^ A mole ratio of 3.6 Fe/Mn was determined by ICP–OES for these MnFe_2_O_4_ NPs. Due to the enhanced magnetization, ferrofluids made by MnFe_2_O_4_ NPs (Figure 1G) responded more strongly to the applied magnetic field when compared with the one formed by Fe_3_O_4_ NPs (Figure 1D).

### Fluorous Ferrofluid Synthesis Using a Biphasic Approach

**Figure 2:**
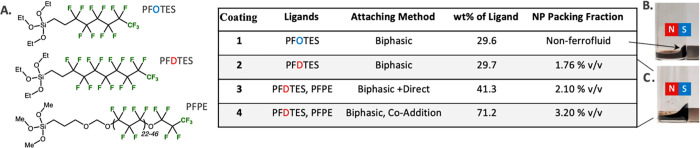
(A) Molecular structure of ligands used to functionalize the NP surface and the summary table of Coatings **1**–**4**, including the corresponded attachment methods, and the experimental results on weight loss (wt %) of ligands conjugated on NPs by TGA, and the NP packing fraction obtained by ICP-OES. (B) Nonferrofluid and (C) ferrofluids in an applied magnetic field. The arrow shows the clump formed due to agglomeration. NP packing fraction was calculated as (volume occupied by the NPs)/(volume in which the NPs are distributed) × 100. The NP volume was based on the iron concentration in the NP suspension measured by ICP-OES.

**Figure 3:**
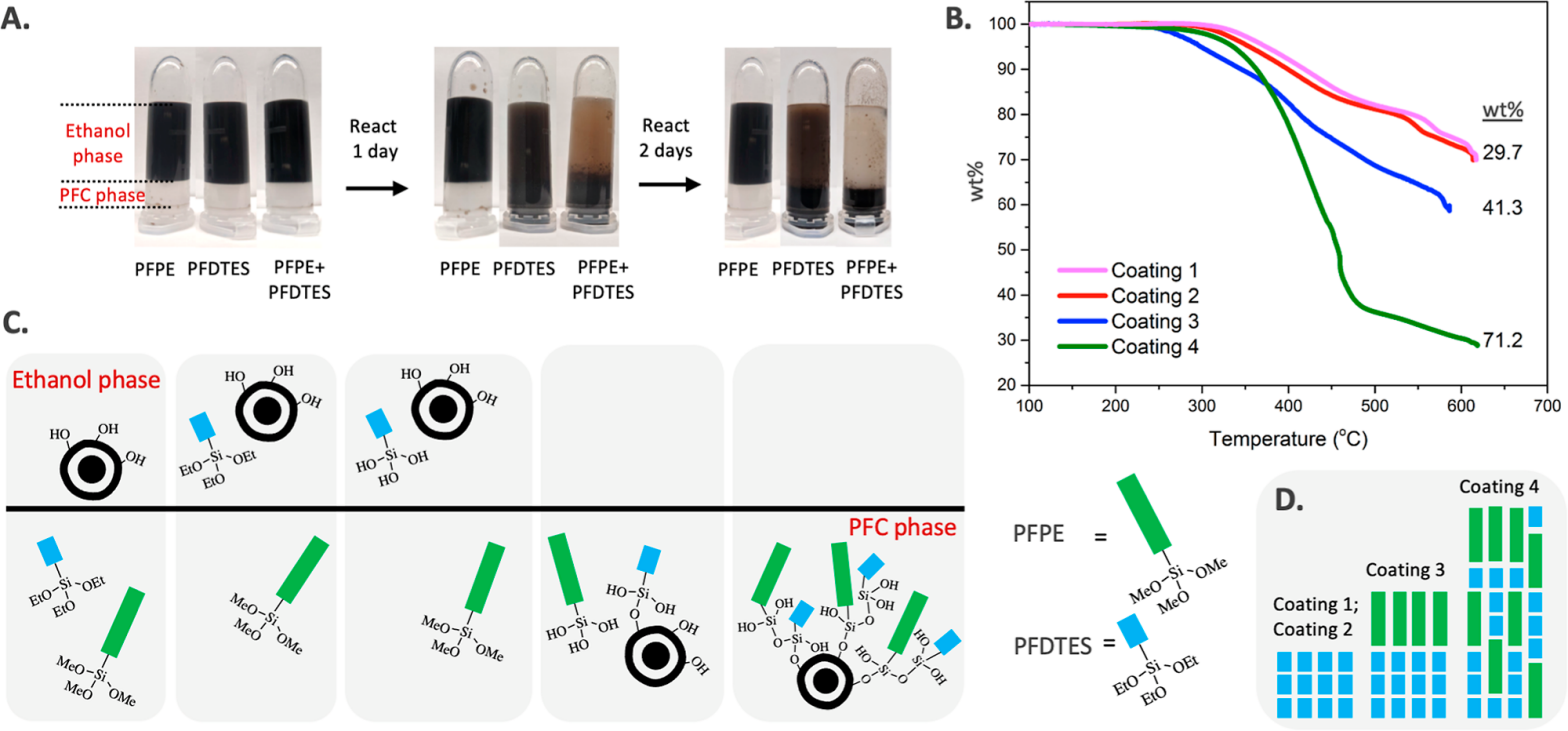
(A) Two-phase system containing an upper ethanol phase with Fe_3_O_4_@SiO_2_ NPs or MnFe_2_O_4_@SiO_2_ NPs (5 mg/mL) and a lower PFC phase containing PFPE, PFDTES, or PFPE + PFDTES after 1 day or 3 days’ mixing. (B) TGA of NPs shows ligand attachment from 29.7 wt % with Coating **1** or Coating **2** to 41.3 wt % with Coating **3**, and to 71.2 wt % with Coating **4**. (C) Schematic illustration of the two-phase system showing that ethanol soluble PFDTES can diffuse through both phases when mixed, facilitating the partition of the NPs into the PFC phase and allowing for the iterative attachment of PFPE onto the NP surface. (D) Schematic illustration of Coatings **1**–**4** on the NP surface, where PFPE was attached via silanol polymerization.

We surveyed three different ligands, perfluorooctyltriethoxysilane (PFOTES, called Coating **1**), perfluorodecyltriethoxysilane (PFDTES, called Coating **2**), and perfluoropolyethertrimethoxysilane with 22–46 repeat units (called PFPE; used in Coatings **3** and **4**). The structures and the full names of each PFC carrier liquid can be found in Figure 2A and Table S1. Notably, all three silanes contained methylene spacers to shield the silane from the electron-withdrawing effects of the fluorinated tails. Both PFOTES and PFDTES have heavily fluorinated tail groups yet are miscible with ethanol. A biphasic approach was employed to bond the fluorous ligands to the surface silanol groups on Fe_3_O_4_@SiO_2_ or MnFe_2_O_4_@SiO_2_ NPs. The ethanol solution containing Fe_3_O_4_@SiO_2_ or MnFe_2_O_4_@SiO_2_ and the PFC solvent containing the desired fluorous ligands formed a two-phase system (Figure 3A). A small amount of water was added to the ethanol phase to promote the hydrolysis of perfluoroalkyl silanes. When the two phases were mixed, ethanol-soluble fluorous ligands diffused through both phases, became hydrolyzed due to the presence of water in the ethanol phase, and underwent condensation with the abundant –OH groups on the Fe_3_O_4_@SiO_2_ or MnFe_2_O_4_@SiO_2_ NPs to form covalent siloxane Si–O–Si bonds. This biphasic approach helps prevent self-condensation of the silanes due to the localization of hydrolyzed silanes near the –OH groups on NPs and can be used to monitor the attachment of fluorous silanes on NPs. When the Fe_3_O_4_@SiO_2_ or MnFe_2_O_4_@SiO_2_ NPs were functionalized with enough fluorous silanes, they became soluble in the PFC phase, and phase transfer of NPs from the ethanol layer to the PFC layer occurred. Phase transfer upon ligand exchange has also previously been reported for fluorous AuNP synthesis.^25^

The ability of ligands to stabilize the NPs in fluorous solvents is very sensitive to their compositions and structures. For example, the silanes used for Coatings **1** and **2** differ by only one carbon–carbon bond but resulted in fluorous NPs that behaved very differently in the PFC carrier liquid. While neither Fe_3_O_4_@SiO_2_-**1** nor MnFe_2_O_4_@SiO_2_-**1** NPs formed a ferrofluid with the carrier liquid HFE-7700 and agglomeration of NPs was observed (Figure 2B), Fe_3_O_4_@SiO_2_ and MnFe_2_O_4_@SiO_2_ NPs with Coating **2** (Fe_3_O_4_@SiO_2_-**2** or MnFe_2_O_4_@SiO_2_-**2** NPs) formed very stable ferrofluids. We attribute this result to the additional extra interparticle distance created by Coating **2** compared to Coating **1**. The fluorous ferrofluids with Coating **2** displayed both magnetic property and liquid behavior and responded to an applied external field without agglomeration or coagulation (Figures 2C and S3). A similar weight loss (wt %) of Coating **1** to that of Coating **2**, which was obtained by thermogravimetric analysis (TGA), further confirmed that the difference between NPs coated with **1** and **2** was due to the molecular structure of the ligand and not the density installed on the NPs.

### Fluorous Ferrofluids with NPs Coated with High-Molecular-Weight PFPE with the Assistance of Lower Molecular Weight PFDTES

The ligands on the outer surface of the NPs provide a surface chemical composition that is largely associated with the compatibility between magnetic NPs and the liquid carrier.^20^ To test this, we used PFPE, a fluorous perfluoropolyether, that has a molecular weight 10 times larger and a linear molecular length that is approximately 5 times longer than PFDTES. We tried multiple conditions using the biphasic approach to attach PFPE on NPs’ surface (listed in Table S2; see Supporting Information for details). However, none of them facilitated the phase transfer of magnetic NPs to the PFC phase. This is likely due to the insolubility of PFPE in the ethanol phase, which restricts PFPE from having direct contact with water molecules and –OH groups on Fe_3_O_4_@SiO_2_ NPs in the ethanol phase.

A direct coating approach was first used to attach PFPE to Fe_3_O_4_@SiO_2_ NPs. Despite the fact that fluorous ferrofluids were attained using the direct coating approach, aggregation was observed, which resulted in a unreliable DLS result. In addition, after drying in air, fluorous ferrofluids synthesized using the direct-coating approach solidified and could not be dispersed back to the PFC oil, indicating the chemical instability of this ferrofluid system.^20^

The successful preparation of ferrofluids using NPs coated with PFDTES (Coating **2**) and the confirmation of rapid silanization between PFPE and Fe_3_O_4_@SiO_2_ NPs in a PFC oil by the direct coating approach suggested that a hybrid of the coating methods could result in higher performing, more stable ferrofluids. Fluorous ferrofluids containing both PFDTES and PFPE were successfully prepared via a two-step procedure: biphasic coating with PFDTES followed by direct coating with PFPE. Briefly, after Fe_3_O_4_@SiO_2_-**2** NPs were phase-transferred to the PFCs oil via the biphasic approach, the Fe_3_O_4_@SiO_2_-**2** NPs were washed and redispersed in PFC solvent. This Fe_3_O_4_@SiO_2_-**2** NP suspension was mixed with PFPE in a direct-coating step to generate Fe_3_O_4_@SiO_2_-**3** NPs containing both ligands. The successful attachment of PFPE was confirmed by TGA which shows an increase in weight loss from 29.7 to 41.3 wt % following the attachment of PFPE via the direct oligomerization of silanols (Figure 3B). The DLS shows an average NP size of 117.5 nm with a PDI of 0.161 for NPs with Coating **3**, implying the successful preparation of monodispersed NPs (Figure S1). Next, we compared the solubility of NPs with Coating **2** with that of Coating **3** NPs by measuring the NP packing fraction, defined as the volume occupied by the NPs divided by the volume the NPs are distributed.^31^ The volume of NPs, assuming all NPs contain a single core, was determined by measuring the iron concentration in each saturated NP suspension using ICP-OES. The NP packing fraction of ferrofluids increased from 1.76% v/v to 2.10% v/v after the PFPE attachment, suggesting that introduction of a high-molecular-weight silane ligand played a crucial role in improving compatibility between fluorous Fe_3_O_4_@SiO NPs and PFC solvents.

The experiments described above show that high-MW PFPE can be installed on the surfaces of Fe_3_O_4_@SiO_2_ NPs through covalent Si–O–Si bonds with the active –OH NP surface and with the silanol layer of the pregrafted ligands. With this knowledge in mind, we concurrently added both low-MW PFDTES and high-MW PFPE to the two-phase system (Figure 3A) to coat the surface of the Fe_3_O_4_@SiO_2_ NPs. The presence of both PFDTES and PFPE during Fe_3_O_4_@SiO_2_ NPs surface functionalization increased the wt % of ligands conjugated onto the NP surface. NPs underwent isolation from 29.7 wt % with just PFDTES alone (Coating **2**) to 41.3 wt % with PFDTES and then PFPE (Coating **3**) and to 71.2 wt % with both PFDTES and PFPE (Coating **4**), as shown by TGA (Figure 3B). The enhanced increase in total conjugated ligands also improved the solubility of Fe_3_O_4_@SiO_2_-**4** NPs (3.20% v/v) in PFC solvent. The ethanol-soluble PFDTES facilitated the partition of the NPs into the PFC phase, allowing fast and iterative attachment of PFPE onto the active NP surface, as shown in the schematic illustration (Figure 3C). Therefore, this coaddition approach accelerated the phase transfer (ethanol to PFC) process and maximized the chance of PFPE to condense with the remaining silanol sites on the NP surface before these silanol sites are fully reacted with, and covered by, free PFDTES present in the reaction. Coating **3** can be attributed to the polymerization of the silanols of PFDTES and PFPE (Figure 3D). For Coating **4**, low-MW PFDTES not only brought both the Fe_3_O_4_@SiO_2_ NPs and the absorbed water residues to the PFCs phase but also created additional silanol sites for PFPE to attach via polymerization. In addition, smaller sized PFDTES can access the free spaces in the outermost fluorous ligand layer, facilitating more silane attachments via the Si–O–Si polymerization. It can also be seen that when the amount of PFDTES in the coaddition biphasic approach was decreased, the weight loss of ligands conjugated onto the NP surface went down to 69.3 wt % (Fe_3_O_4_@SiO_2_-**9** NPs, Figure S5). Alongside verification that PFDTES assists with the attachment of PFPE on the NP surface, we validated that PFDTES plays an essential role in the attachment of PFPE by carrying out an experiment with the same conditions mentioned above but in the absence of PFDTES, where Figure 3A shows that all Fe_3_O_4_@SiO_2_ NPs remained in the ethanol phase after 3 days of mixing.

The resulting fluorous Fe_3_O_4_@SiO_2_ ferrofluids were further characterized by DLS, FTIR, and TEM. DLS of Fe_3_O_4_@SiO_2_-**4** NPs showed a monodisperse population (PDI = 0.226) and an average NPs size that agrees with the length of ligand used, where the functionalization including polymeric silane PFPE resulted in a significant size increase of Fe_3_O_4_@SiO_2_-**4** NPs (105 nm by DLS, Figure S1). TEM images showed that the Fe_3_O_4_@SiO_2_ NPs stayed intact after fluorous functionalization (Figure S6). In the FTIR spectrum (Figure S7), the two peaks at ν = 802 and 1101 cm^–1^ arise from the Si–O–Si symmetric and asymmetric stretching, respectively, in the unfunctionalized Fe_3_O_4_@SiO_2_ NPs and the peak at ν = 950 cm^–1^ corresponds to the Si–OH groups. FTIR spectra of all fluorous NPs have two peaks that appear at ν = 1149 and 1207 cm^–1^ due to C–F stretching, confirming the presence of C–F bonds attached to the surface of the Fe_3_O_4_@SiO_2_ NPs.

To further validate that both PFDTES and PFPE are chemically grafted onto the NP surface, we used the biphasic approach to prepare the negative control Fe_3_O_4_@SiO_2_-**10** NPs. Previously, we observed that Coating **10** is sensitive to ultrasonication. When Fe_3_O_4_@SiO_2_-**10** NP was suspended in the PFC phase, they would gradually transfer back to ethanol phase under ultrasonication due to the loss of ligands that were originally on the NP surface (Figure S8A). The instability of coating **10** was confirmed by the TGA measurement. While loosely attached Coating **10** decomposed at a temperature of 150 °C, Coating **4** only started to decompose when a temperature of >300 °C is reached, indicating the chemical stability of the attached PFDTES and PFPE via the Si–O–Si bond formation (Figure S8B). Interestingly, stability of Coating **10** can be improved if they were installed on the NP surface in the presence of PFDTES (coaddition biphasic approach, Fe_3_O_4_@SiO_2_-**11** NPs). Under ultrasonication, Fe_3_O_4_@SiO_2_-**11** NPs remained in the PFCs phase (Figure S8A) and TGA of Coating **11** showed no decomposition at 150 °C (Figure S8B). This result further verifies that the coaddition of high-MW surfactants with low-MW surfactants facilitates the attachment of high-MW PFPE and produces stable and nonionic fluorinated ferrofluids.

**Figure 4:**
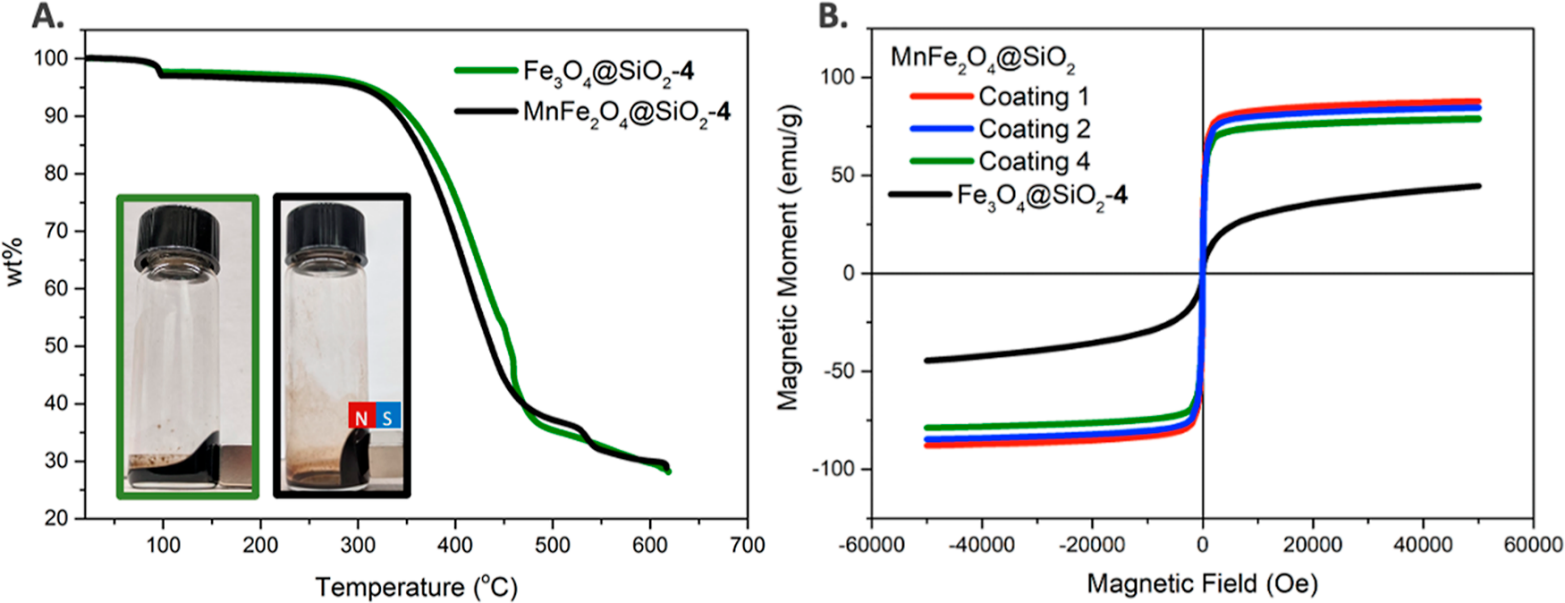
(A) TGA shows a 71.2 wt % of conjugated ligands for both Fe_3_O_4_ NPs and MnFe_2_O_4_ NPs with Coating **4**. The inset shows that ferrofluids (3.20% v/v) made by MnFe_2_O_4_@SiO_2_-**4** NPs respond more strongly to the applied magnetic field (a rare earth magnet) when compared with the one formed by Fe_3_O_4_@SiO_2_-**4** NPs with the same concentration. (B) SQUID M–H loop of MnFe_2_O_4_@SiO_2_-**1**, MnFe_2_O_4_@SiO_2_-**2**, MnFe_2_O_4_@SiO_2_-**4**, and Fe_3_O_4_@SiO_2_-**4** NP suspension (1% v/v). M–H measurements were performed at a temperature of 300 K.

The fluorous ferrofluids using MnFe_2_O_4_ NPs were generated via the same coaddition silanization process and are denoted as MnFe_2_O_4_@SiO_2_-**4** NPs (164 nm by DLS, Figure S1A). TGA shows that for Coating **4**, the mass of the ligands conjugated on MnFe_2_O_4_@SiO_2_ is similar to that of Fe_3_O_4_@SiO_2_ (Figure 4A). Due to the enhanced magnetization, at maximum solubility in PFC solvent, ferrofluids made by MnFe_2_O_4_@SiO_2_-**4** NPs respond more strongly to the applied magnetic field when compared with the one formed by Fe_3_O_4_@SiO_2_-**4** NPs (Figure 4A, inset). The magnetic properties of all fluorous ferrofluids made using MnFe_2_O_4_ NPs, including MnFe_2_O_4_@SiO_2_-**1** NPs, MnFe_2_O_4_@SiO_2_-**2** NPs, and MnFe_2_O_4_@SiO_2_-**4** NPs, were measured, and all of them continued to show superparamagnetic responses. The magnetization hysteresis (M–H) loop (Figure 4C) shows almost no coercive force, validating the superparamagnetic properties of MnFe_2_O_4_ NPs. The saturation magnetization of the ferrofluid formed by MnFe_2_O_4_@SiO_2_-**4** NPs is 82.6 ± 7.9 emu/g, higher than that of Fe_3_O_4_@SiO_2_-**4** NPs (41.6 ± 3.5 emu/g) as shown in Figure 4B.

### Effects of Silica Shell Coatings and Thickness on Magnetization

Semiquantitative comparisons of the attraction of the ferrofluids to a magnet, as shown above, provide a fast and simple way of identifying ferrofluid behavior and relative strengths of the attraction of compositions using different particles. In this section, we utilize two quantitative methods to evaluate the “strengths”: constant temperature saturation magnetization and temperature-dependent magnetization at constant fields.

The constant temperature magnetization curves of ferrofluids at room temperature (300 K) containing coated silica-encased MnFe_2_O_4_ with Coatings **1**, **2**, and **4** shown in Figure 4B provide quantitative magnitudes of the magnetization of the ferrofluids and explain why the ferrofluids made using Fe_3_O_4_ are less strongly attracted to a neodymium magnet than the MnFe_2_O_4_-based ferrofluids. It is interesting to note that Coating **1** (the thinnest) appears to give rise to the highest saturation magnetization and Coating **4** (the thickest) results in the smallest saturation magnetization, but the values are within experimental uncertainty of each other.

The temperature dependence of the magnetization of the NPs in ferrofluids on the applied magnetic field is strongly dependent on the attractive interactions between the NPs and the PFC carrier liquid and to a lesser extent on the interparticle interactions. The effect of these interactions can be observed with temperature-dependent magnetization (M–T) measurements. The strength of these interactions can be adjusted by synthesizing different size coatings. Therefore, we took a closer look at the effects of Coatings **1**, **2**, and **4** on the ferrofluids to compare (a) the magnetic behavior between nonferrofluids (Coating **1**) and ferrofluids (Coatings **2** or **4**) and (b) the differences in the effects caused by thinner (Coating **2**) vs thicker (Coating **4**) fluorous layers.

**Figure 5:**
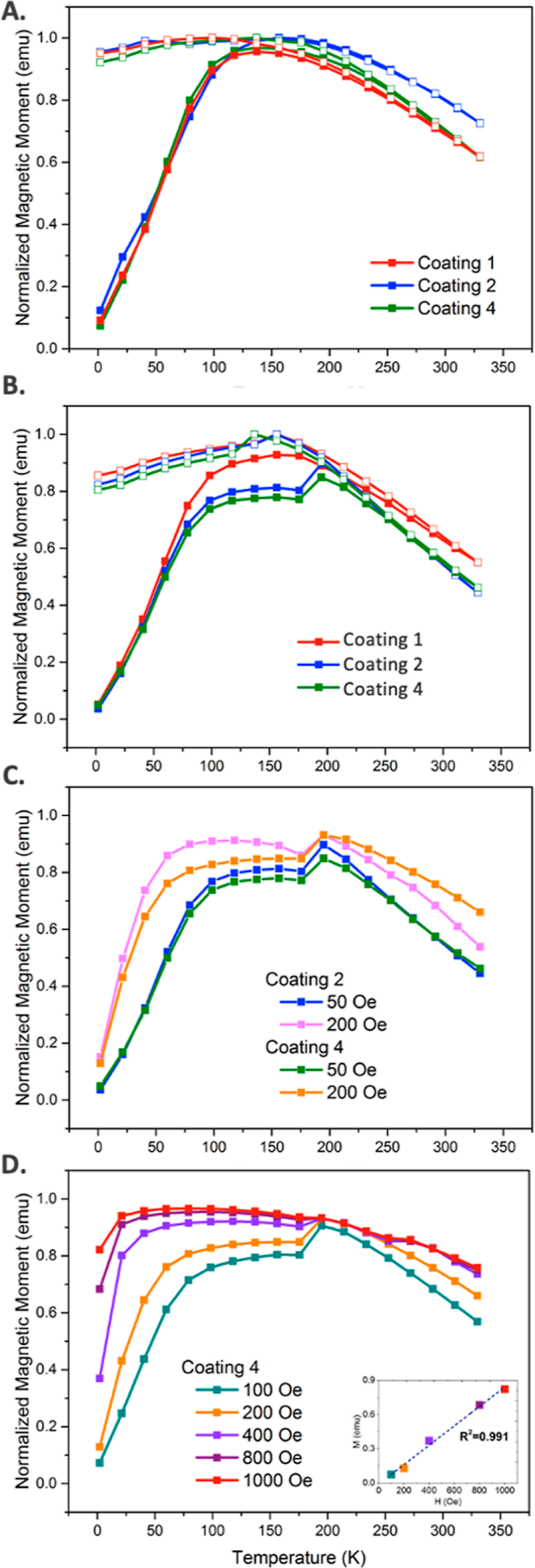
(A) ZFC (filled circles) and FC (unfilled circles) modes of magnetic NPs with Coatings **1**, **2**, and **4** in powder form. (B) ZFC curve of the temperature-dependent magnetization (M–T) of Coatings **1**, **2**, and **4** under an applied magnetic field of 50 Oe. (C) ZFC curve comparison between Coatings **2** and **4** and between an applied magnetic field of 50 and 200 Oe. (D) ZFC curves of Coating **4** under an applied magnetic field of 100, 200, 400, 800, and 1000 Oe. Inset shows the linear fitting of magnetization (M) vs magnetic field strength (H). NP packing fraction = 1% v/v. (B–D) are in liquid form.

The temperature dependences of the magnetic properties of the NPs themselves in powder form in a field of 50 Oe are shown in Figure 5A. As expected, the magnetic moments of the zero-field cooled (ZFC) particles increase with increasing temperature until reaching the blocking temperature (*T*_B_) of 140 K after which the moments decrease. The three curves are very similar at all temperatures.

In contrast, the ZFC curves of the particles in the PFC solvent at a fixed NP concentration (1% v/v) are markedly different, as shown in Figure 5B. The first important difference is the behavior as the temperature increases and approaches *T*_B_. The slopes of the curves are flattened with the largest effect evident in the curve for the ferrofluid that contains particles with Coating **4** and the least effect on the curve of the nonferrofluid suspension of particles with Coating **1**. The plateau is in the temperature range where the system is a rigid solid or transitioning to a “mixed state” and the NPs cannot move easily.^32,33^ The second prominent difference is the abrupt rise at about 200 K for the ferrofluids containing particles with Coatings **2** and **4**. The pour point (*T*_pour_) of the PFC solvent HFE-7700 is 193–223 K, where the system at temperatures above *T*_pour_ can flow like a fluid. Similar behavior has been observed in the ZFC-FC curves of Fe_3_O_4_ NPs in dodecane ferrofluids at their pour point.^32^

The appearance of the distinct flattening or plateau for Coatings **2** and **4** is attributable to the restraint of the NPs’ movement to align their magnetic fields with the applied field resulting from the strong interactions between ligands and the surrounding solvent molecules that restrain the particles’ movements.^32^ The magnetization involving Coating **1**, the thin coating that does not give rise to ferrofluidic behavior, behaves almost like the particles with no solvent (Figure 5A) because the particles interact less strongly with the solvent and are less constrained. The motion of particles to align their magnetic moment with the applied magnetic field is sometimes called Zeeman alignment in the literature.^25^

To further test the effect of the magnetic field strength on magnetization behavior resulting from particle alignment, we increased the field strength from 50 to 200 Oe. The high-field ZFC curves are plotted together with the 50 Oe curves for comparison in Figure 5C. At the higher field, the differences between the Coating **2** and **4** curves are increased with both curves showing faster increases in response to the increased field strength and that of Coating **2** rising significantly faster than that of Coating **4**. The plateau for Coating **2** is higher and broader. These effects are a further indication of the stronger interaction of the solvent with Coating **4** compared to that of Coating **2**. The sharp rise at 177 K in the ZFC curves is still present for both Coating **2** and Coating **4** due to the decrease in the constraint of the NPs’ motions above the pour point temperature.

We further investigated the strong interaction between Coating **4** and the suspension media by taking the ZFC measurements as a function of field strength (Figure 5D). We found that while the dip at 177 K is still present in the magnetization curves taken at applied fields up to 400 Oe, a relatively smoothly decreasing curve was obtained when fields of 800 and 1000 Oe were applied (Figure 5D). These results may be a result of the stronger dipolar alignment forces in high fields (>800 Oe) overriding the interactions between NPs and carrier liquid, allowing magnetic moments of NPs to achieve the most stable state quickly. It can be seen that even at 2 K, the magnetization increases as the applied magnetic field increases, and the maximum magnetization of the ZFC curves was reached in the lower temperature range (Figure 5D). Collectively, our results demonstrate that the size and thickness of the ligands tailor the interactions between the NPs and the molecules of the carrier liquid.

## Conclusions

In this study, we developed a biphasic ligand attachment method and showed that both the selected ligands and our surface functionalization strategies are essential for synthesizing stable nonionic fluorous ferrofluids. We demonstrated that a thin silica layer around the superparamagnetic core allows the surface functionalization of fluorous ligands through stable covalent chemistry. We found that the low-MW ligands can assist with the installation of high-MW ligands on the NP surface, allowing us to systematically control the outer surfactant layer of the magnetic NPs and therefore the tailoring of the interparticle distance. Additionally, the use of constant temperature and ZFC magnetization curves for the analysis of interparticle dipole forces and the interplay of interactions between the NPs and the liquid carrier paves the way to optimizing ferrofluids for both biomedical and engineering applications.

## Experimental Section

### Materials and Chemicals

The chemicals used include iron(III) chloride hexahydrate (FeCl_3_·6H_2_O) (≥98%, Aldrich), iron(III) acetylacetonate (Fe(acac)_3_, 97%), manganese(II) acetylacetonate (Mn(acac)_2_), sodium oleate (99%, TGI), oleic acid (90%, Aldrich), oleylamine (70%, Sigma), 1,2-dodecanediol (90%, Sigma), Igepal CO-520 (Sigma), tri-*n*-octylamine (95%, Fisher), benzyl ether (98%), tetraethyl orthosilicate (TEOS) (≥99%, Sigma), 1*H*,1*H*,2*H*,2*H*-perfluorooctyltriethoxysilane (PFOTES, 97%, Sigma), 1*H*,1*H*,2*H*,2*H*-perfluorodecyltriethoxysilane (PFDTES, 97%, Sigma), 1*H*,1*H*,2*H*,2*H*-perfluorodecyltrimethoxysilane (PFPE, 20% in fluorinated hydrocarbon, MW = 4000–8000 g/mol, 22–46 repeat units, Gelest), silane PFPE_NH_ (MW = 989 g/mol, Surfactis Technologies), ammonia solution (NH_4_OH, 28–30%), hexane (≥98.5%, Fisher), cyclohexane, and chloroform (99.9%, EMD), HFE-7700, HFE-7200, HFE-7100, perfluorohexane (PFH, 3M), and perfluorooctane (PFO, 3M).

### Characterizations

TEM images were acquired on a Tecnai T12 Quick CryoEM and CryoET (FEI) with an operating voltage of 120 kV. The sample was prepared by dropping a suspension (0.2 mg/mL, 5 μL) of NPs in hexane, ethanol, or HFE-7100 on a 200-mesh carbon-coated copper grid, followed by solvent evaporation at room temperature. DLS measurements were acquired on a ZetaSizer Nano (Malvern Instruments Ltd., Worcestershire, U.K.) in ethanol for Fe_3_O_4_@SiO_2_ and in HFE-7700 for Fluorous Fe_3_O_4_@SiO_2_. Fourier-transform infrared spectroscopy (FTIR) analyses were performed on an FTIR spectrometer (JASCO FT/IR-420) in the range of 4000–400 cm^–1^. TGA was performed on a PerkinElmer Pyris Diamond TG/DTA machine under air flow. Sample was loaded in aluminum pans, and the data were recorded from 50 to 100 °C at a scan rate of 20 °C/min, maintained at 100 °C for 30 min to remove water residuals, 100 to 600 °C at a scan rate of 15 °C/min, and finally maintained at 600 °C for 1 h. An empty aluminum pan was used as the reference. A superconducting quantum interference device (SQUID) Quantum Design MPMS3 magnetometer was used to confirm the superparamagnetic behavior of fluorous ferrofluids. The field-dependent magnetization curves were measured at 300 K, and the temperature-dependent magnetization curves were measured in an applied magnetic field of 50, 100, 200, 400, 800, or 1000 Oe. The ferrofluid was sealed inside a glass capillary tube (the suspension has an aspect ratio of <6), and the capillary tube was secured inside the straw. Inductively coupled plasma optical emission spectroscopy (ICP-OES) using a Shimadzu ICPE-9000 instrument was carried out to quantify the iron/manganese ratio of MnFe_2_O_4_ NPs and to determine the iron concentration in each saturated NP suspension for solubility comparison. 10 mL of Aqua regia was used to digest each sample for 3 h. Upon evaporation of aqua regia overnight, the sample was diluted with 10 mL of 2.5% nitric acid.

### Synthesis of Fe_3_O_4_ NPs

Fe_3_O_4_ NPs were synthesized by a modified thermal decomposition method.^27^ FeCl_3_·6H_2_O (2 mmol) and sodium oleate (6 mmol) were dissolved in 14 mL of a solvent mixture composed of ethanol, Millipore water, and hexane (volumetric ratio of 4:3:7). After refluxing at 70 °C for 4 h, the solution was transferred to a separatory funnel. The top layer containing the Fe-oleate was washed with water and ethanol (3 × 5 mL). After evaporating hexane at 70 °C overnight, 3.2 mmol oleic acid and 10 mL of tri-*n*-octylamine were added to the Fe-oleate complex precursor. The mixture was degassed with N_2_ under stirring for 30 min at room temperature before heating to 200 °C at a heating rate of 3 °C/min^–1^. After staying at 200 °C for 2 h, the mixture was heated to 330 °C at the same heating rate. The mixture was allowed to reflux and age at 330 °C for 1 h before cooling to room temperature under N_2_. Afterward, a mixture of ethanol and acetone (1:1) was added to precipitate the black product. The precipitate was collected by centrifugation, washed three times with ethanol, and redispersed in hexane containing 50 μL of oleic acid.

### Synthesis of MnFe_2_O_4_ NPs

MnFe_2_O_4_ NPs were synthesized by a modified seed-mediated thermal decomposition method involving a high-temperature reaction of 2 mmol Fe(acac)_3_ and 1 mmol Mn(acac)_2_ with 1,2-hexadecanediol (10 mmol), oleic acid (6 mmol), and oleylamine (6 mmol) in 15 mL of benzyl ether. All other procedures and reaction temperatures were the same as that of Fe_3_O_4_ NP synthesis. Finally, the resulting 8.4 nm MnFe_2_O_4_ NPs were dispersed in 20 mL of hexane containing 50 μL of oleic acid. The larger 15.4 nm MnFe_2_O_4_ NPs were synthesized by growing MnFe_2_O_4_ on 8.4 nm MnFe_2_O_4_ NPs. Briefly, 7 mmol Fe(acac)3, 3.5 mmol Mn(acac)2, 35 mmol 1,2-dodecanediol, 7 mmol of oleic acid, and 7 mmol oleylamine were dissolved in 20 mL of benzyl ether in a 100 mL three-necked flask. The 8.4 nm MnFe_2_O_4_ NPs (330 mg, 20 mg/mL) were added to the reaction mixture, which was then heated to 100 °C to evaporate hexane (cease of bubbling indicates the completion of hexane evaporation). The rest of the synthesis procedures were the same as that of 8.4 nm MnFe_2_O_4_ NP synthesis.

### Synthesis of Fe_3_O_4_@SiO_2_ NPs

Fe_3_O_4_@SiO_2_ NPs were synthesized based on a published reverse-microemulsion approach with some modifications.^24^ In a typical synthesis of Fe_3_O_4_@SiO_2_ NPs, 0.6 g of Igepal CO-520 was dispersed in cyclohexane (19.4 mL) and sonicated for 20 min in a 50 mL three-necked flask. Then, oleic acid–capped Fe_3_O_4_ NPs dispersed in cyclohexane (9 mL) were added into the preprepared cyclohexane/Igepal CO-520 mixture. After 15 min of sonicating and 4 h of magnetic stirring, 140 μL of NH_4_OH (28–30%) was added to the mixture, and the system was sealed and stirred for another 3 h. 100 μL of TEOS was injected into the mixture dropwise, and the system was kept under magnetic stirring for 36 h at room temperature before adding 5 mL of methanol to disrupt the emulsions.

### Synthesis of Perfluoro Fe_3_O_4_@SiO_2_-**1**, Fe_3_O_4_@SiO_2_-**2**, and Fe_3_O_4_@SiO_2_-**10** NPs

Biphasic exchange method was implemented for the PFDTES attachment. In an Eppendorf tube, 500 μL of PFO and 1 mL of ethanol of Fe_3_O_4_@SiO_2_ NP suspension (5 mg/mL) form a two-phase system. PFDTES was added directly into the two-phase system, followed by the addition of aliquot of water (volume ratio of ethanol/PFPES/water = 20:1:0.5 and 40:1:0.5 for first and second addition, respectively.) The suspension was allowed to rotate for 2 days to bring the Fe_3_O_4_@SiO_2_-**2** NPs down to the perfluoro phase. Fe_3_O_4_@SiO_2_-**2** NPs were washed with a solvent mixture (ethanol/HFE-7200 = 5:1) two times before being resuspended in HFE-7700.

The same procedure was performed for synthesizing Fe_3_O_4_@SiO_2_-**1** NPs except that PFOES was used as the surfactant. Synthesis of Fe_3_O_4_@SiO_2_-**10** NPs was the same except that PFPE containing an amide (PFPE_NH_) was used as the surfactant. The same procedures were performed for the synthesis of MnFe_2_O_4_@SiO_2_-**1** and MnFe_2_O_4_@SiO_2_-**2** NPs.

### Synthesis of Fe_3_O_4_@SiO_2_-**3** NPs (Biphasic + Direct Coating)

Fe_3_O_4_@SiO_2_ NPs were first transferred to the perfluoro phase with the same procedure as that of Fe_3_O_4_@SiO_2_-**2** NPs. The washed Fe_3_O_4_@SiO_2_-**2** NPs were redispersed in 200 μL of HFE-7100 and added with 50 μL of PFPE. The remaining procedures for three attachments are the same as that of Fe_3_O_4_@SiO_2_-**2** NPs. The final Fe_3_O_4_@SiO_2_-**3** NPs were resuspended in HFE-7700.

### Synthesis of Fe_3_O_4_@SiO_2_-**4**, Fe_3_O_4_@SiO_2_-**9**, and Fe_3_O_4_@SiO_2_-**11** NPs

Biphasic exchange method was implemented for coattachment of PFDTES and PFPE. In an Eppendorf tube, 500 μL of PFH (containing 200 μL of PFDTES and 50 μL and PFPE) and 1 mL ethanol of Fe_3_O_4_@SiO_2_ NP suspension (5 mg/mL) form a two-phase system. The system was added with an aliquot of water (volume ratio of ethanol/PFDTES/water = 20:1:0.5). In the next day, second addition of PFDTES and water (ethanol/PFDTES/water = 40:1:0.5) was carried out. The suspension was allowed to rotate for another 2 days to allow complete surface coverage and to bring the PFDTES and PFPE-functionalized NPs down to the perfluoro phase. After phase transfer, Fe_3_O_4_@SiO_2_-**4** NPs were washed with the solvent mixture (ethanol/HFE-7200 = 5:1) two times before being resuspended in HFE-7700. The same procedures were performed for the synthesis of MnFe_2_O_4_@SiO_2_-**4** NPs. Synthesis of Fe_3_O_4_@SiO_2_-**9** NPs was the same except that 100 μL, instead of 200 μL, of PFDTES was used. Synthesis of Fe_3_O_4_@SiO_2_-**11** NPs was the same except that 200 μL of PFPE-containing amide (PFPE_NH_) was used.

### Synthesis of Fe_3_O_4_@SiO_2_-**5** or Fe_3_O_4_@SiO_2_-**6** Nanoparticles by Direct Coating

Solvent exchange (centrifugation/redispersion) was first performed to transfer Fe_3_O_4_@SiO_2_ NPs from ethanol to HFE-7100 because PFPE is not soluble in ethanol. 50 μL of PFPE was added directly to the Fe_3_O_4_@SiO_2_ NP suspension (20 mg/mL). The suspension was allowed to sonicate for 1 h, followed by overnight rotation to make sure the NP surface is covered by PFPE. The Fe_3_O_4_@SiO_2_-**5** NPs were washed with the solvent mixture (ethanol/HFE-7200 = 5:1) two times before being resuspended in HFE-7700. The same procedure was performed for synthesizing Fe_3_O_4_@SiO_2_-**6** NPs except that 50 μL of PFDTES was added to NPs’ suspension.

### Synthesis of Fe_3_O_4_@SiO_2_-**7** or Fe_3_O_4_@SiO_2_-**8** Nanoparticles by Direct Coating

The washed Fe_3_O_4_@SiO_2_-**5** NPs or Fe_3_O_4_@SiO_2_-**6** NPs were resuspended in HFE-7100 containing 50 μL of PFDTES or PFPE, respectively. The suspension was placed in a sonication bath for 1 h and allowed to undergo silanization under rotation overnight at room temperature.

## Supporting Information Available

Ligands used for each coating, different biphasic conditions tested, fluorous ferrofluids, and DLS, TEM, TGA, and FTIR data (PDF)

## Abbreviations

PFCs: perfluorocarbons
NPs: nanoparticles
PFOTE: perfluorooctyltriethoxysilane
PFDTES: perfluorodecyltriethoxysilane
PFPE: perfluoropolyethertrimethoxysilane
