# Supplementary material for: Synthesis of Fluorous Ferrofluids and Effects of the Nanoparticle Coatings on Field- and Temperature-Dependent Magnetizations
Source: Chem Mater. 2023 Sep 29;35(19):7957–66. doi: 10.1021/acs.chemmater.3c01172 (PMC10569041; doi:10.1021/acs.chemmater.3c01172)
Supplement: Supplementary file 1 — cm3c01172_si_001.pdf [file cm3c01172_si_001.pdf]

Supporting Information

Synthesis of Fluorous Ferrofluids and Effects of the Nanoparticle Coatings on Field and Temperature Dependent Magnetizations

*Fang-Chu Lin[1,2], Heidi L. van de Wouw[1,2], Otger Campàs[3], Ellen Sletten[1,2] and Jeffrey I. Zink[1,2]**

Coating	Ligands	wt% of Ligand	Attach Method	HFE-7100	HFE-7200	HFE-7700
1	PFOTES	29.6	Biphasic	no	no	no
2	PFDTES	29.7	Biphasic	no	no	1.76 % v/v
3	PFDTES, PFPE	41.0	Biphasic +Direct	no	no	2.10 % v/v
4	PFDTES, PFPE	69.3	Biphasic, Co-Addition	no	no	3.20 % v/v
5	PFPE	28.9	Direct	N/A	N/A	Aggregation
6	PFDTES	9.8	Direct	N/A	N/A	Aggregation
7	PFPE, PFDTES	31.8	Direct	N/A	N/A	Aggregation
8	PFDTES, PFPE	34.5	Direct	N/A	N/A	Aggregation
9	$PFDTES_{1/2}$, PFPE	57.0	Biphasic, Co-Addition	no	no	2.73 % v/v
10	$PFPE_{NH}$	62.3	Biphasic		US-sensitive	
11	PFDTES, $PFPE_{NH}$	63.5	Biphasic, Co-Addition		Not US-sensitive	

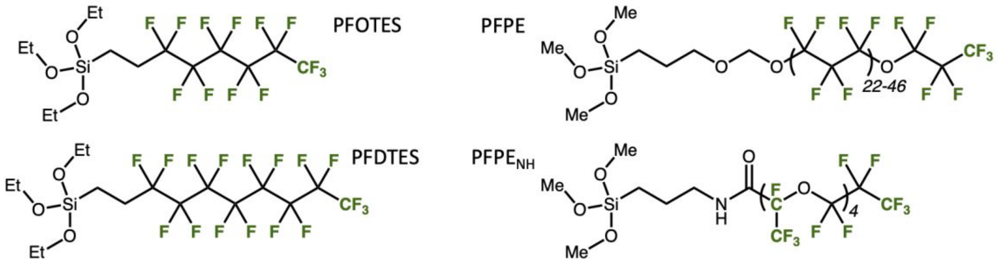

Table S1. Information for Coatings **1-11**, including the ligands used, the corresponding attaching method, the TGA results on weight loss (wt%) of ligands conjugated on NPs, and the perfluoro solvents tested for the synthesis of ferrofluids. The NP packing fraction was only available for ferrofluids. NP packing fraction was calculated as (volume occupied by the NPs)/(volume in which the NPs are distributed)×100. The NP volume was based on the iron concentration in the NP suspension measured by ICP-OES. US = Ultrasound. Below the table is the molecular structure of each ligand.

Upper phase solvent	Ethanol									
Weight of nanoparticles (mg)	6	6	2	6	6	6	3	3	3	6
Lower phase perfluoro solvent	PFH								PFO	PFH
Perfluoro solvent amount (uL)	200						500	200	500	200
Amount of 2 (uL)	20	50	50	100	50	100	100	100	100	100
Water (uL)	100	100	0	0	200	200	100	300	300	100

Table S2. Different biphasic conditions tested to install PFPE on surface of Fe_3O_4@SiO_2 NPs. Upper phase contains 1 mL of solvent. Solvent mixture of Ethanol/HFE-7100/PFH/H_2O (3:1:1:0.1) or Ethanol/HFE-7100/H_2O (3:1:0.1) was also tested. The reactions were allowed to be rotated for 1 week. The silanization of PFPE with -OH groups on NP surface was monitored by the phase transfer of Fe_3O_4@SiO_2 NPs, where Fe_3O_4@SiO_2 NPs remained in the upper phase even with prolonged reaction time (>1week).

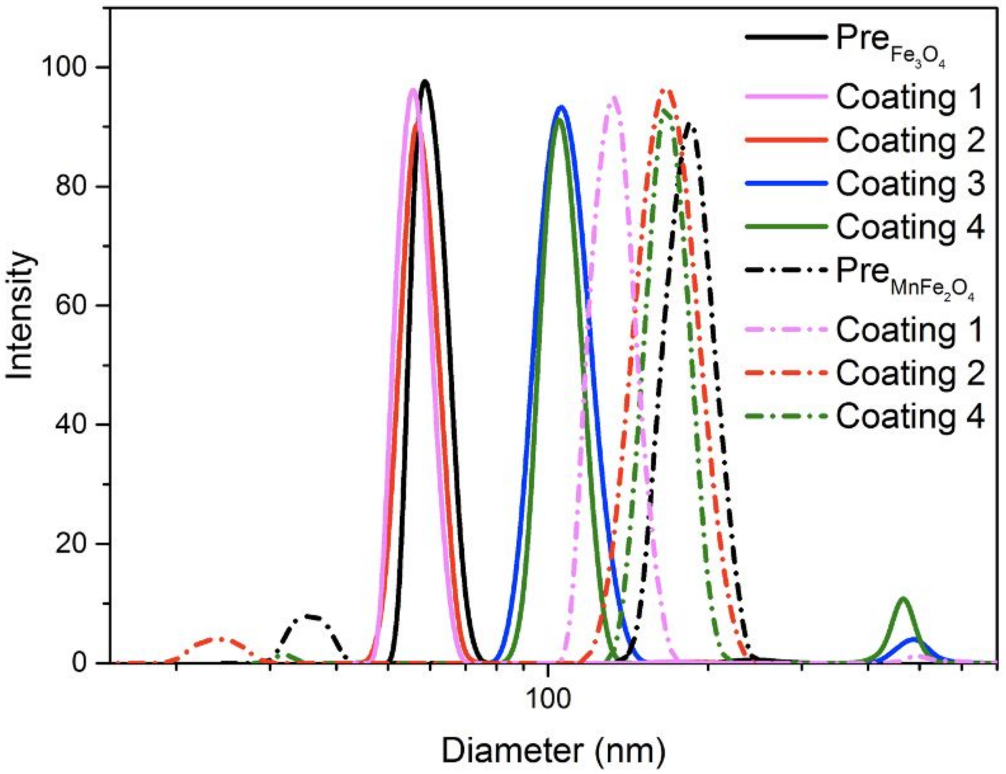

Figures S1. DLS results of Fe_3O_4@SiO_2 NPs and $MnFe_2O_4$@SiO_2 NPs with different fluorous coatings.

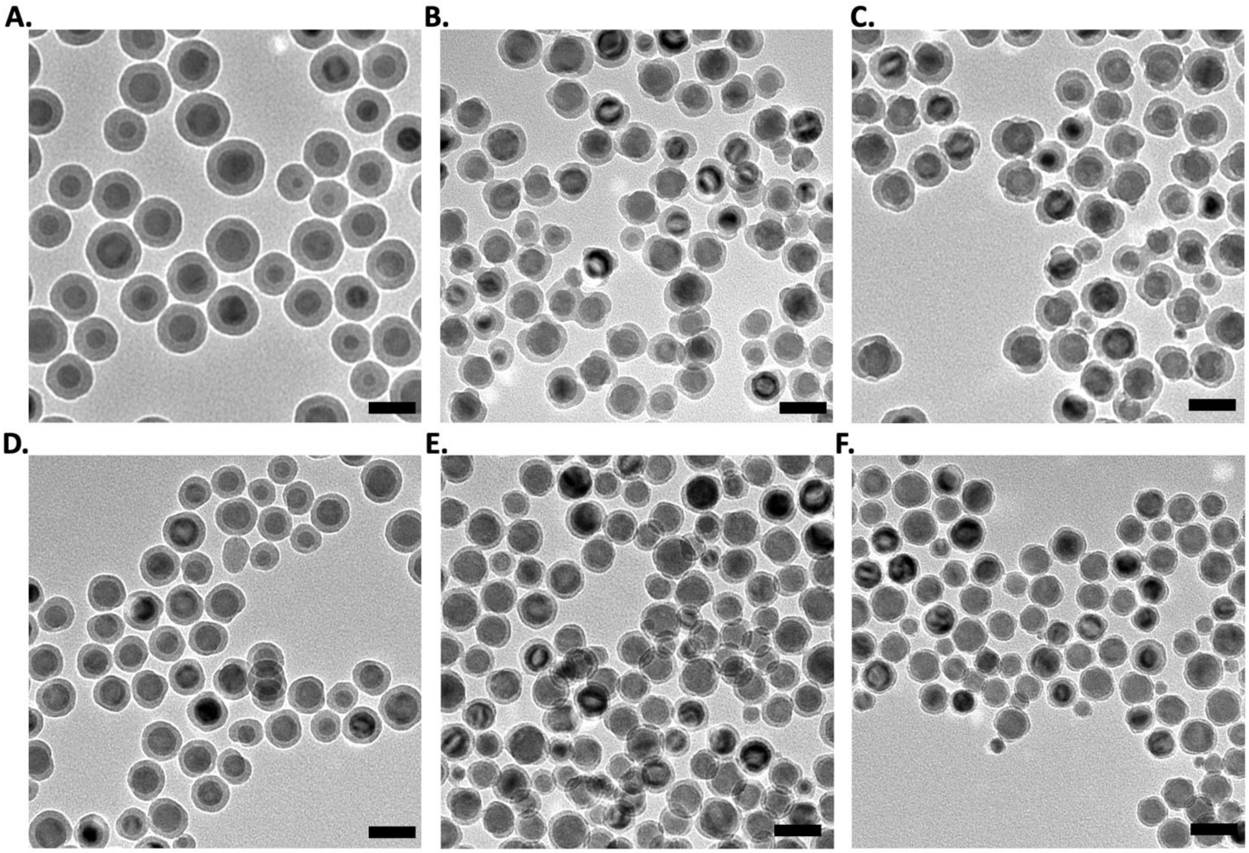

Figure S2. $Fe_3O_4@SiO_2$ NPs with (A) 15 nm silica shell thickness, (B, C) Rough silica surface when only the amount of TEOS was reduced. Silica shell thickness of (D) 10 nm, (E) 5 nm, and (F) 3 nm when both TEOS and ammonia were adjusted. The volumes (μL) of TEOS:NH_4OH ratio in order of A-F are [100:140], [50:140], [25:140], [50:100], [25:100], and [25:70].

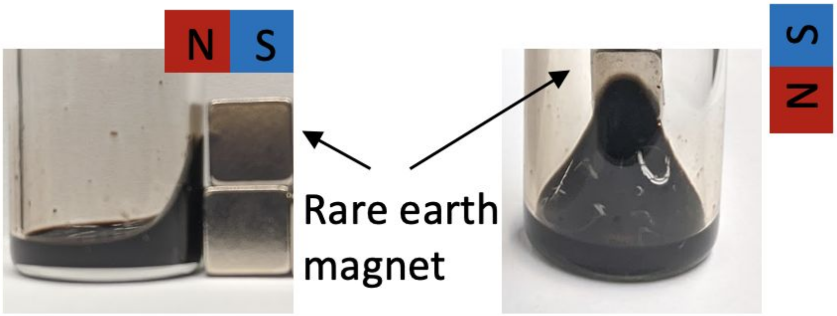

Figure S3. Photos of fluorous ferrofluids ($Fe_3O_4@SiO_2$-**2**) under an external applied magnetic field.

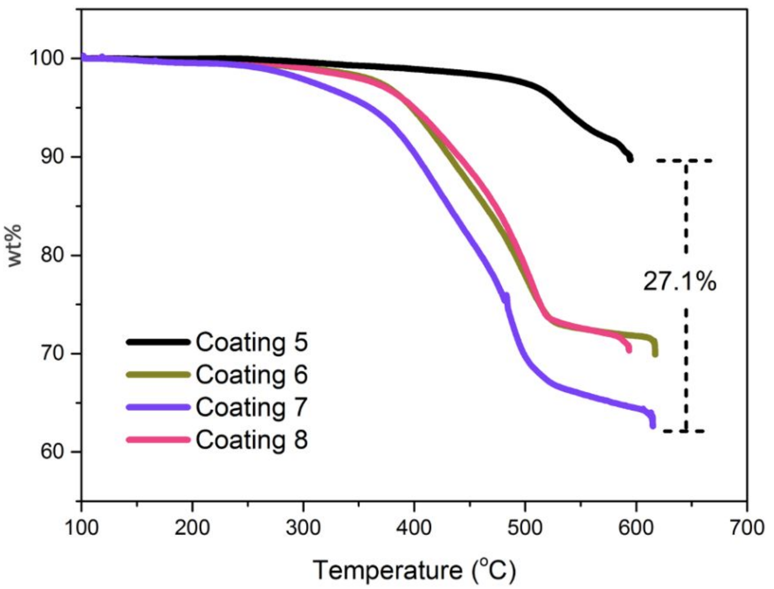

Figure S4. TGA results of ligand attachment, forming Coating **5-8**, via the direct coating approach.

A direct coating approach was used to attach PFPE on Fe_3O_4@SiO_2 NPs, forming Fe_3O_4@SiO_2-**5** NPs. In this approach, Fe_3O_4@SiO_2 NPs were dispersed in PFC oil containing excess amount of PFPE. Condensation between active -OH groups and ligands occurred quickly due to the high reactivity of methyl silanes and this fast process was compared with that of ethyl-silane PFDTES (Fe_3O_4@SiO_2-**6** NPs). As shown by TGA measurements, weight loss of 28.9 wt% and 9.8 wt% was found for the Fe_3O_4@SiO_2-**5** and Fe_3O_4@SiO_2-**6**, respectively (Figure S4). Fe_3O_4@SiO_2-**5** NPs and Fe_3O_4@SiO_2-6 NPs were sequentially added with PFDTES or PFPE, respectively, to generate Fe_3O_4@SiO_2-**7** and Fe_3O_4@SiO_2-**8**. An increase of 27.1% between Coating 6 and 8 indicates the poor surface coverage of Fe_3O_4@SiO_2 NPs by PFDTES via the direct coating due to the slower condensation process of ethyl silanes (Figure S4). The remaining unreactive silanol groups on the surface of Fe_3O_4@SiO_2-**6** NPs allow the sequential attachment of PFPE, resulting in the increased weight loss. In addition, TGA results show that the total silane attachment of Fe_3O_4@SiO_2-**8** NPs is almost 10 wt% more than those of with Coating **7**, suggesting the use of low-MW surfactants for enhancing the attachment of larger-sized surfactants. Note that despite fluorous ferrofluids was attained by using the direct coating approach, aggregation was observed, which resulted in a non-reliable DLS result. In addition, after drying in air, fluorous ferrofluids synthesized by using direct-coating approach solidified and could not redisperse back to the PFCs oil, implying the chemical instability of this ferrofluid system.[1]

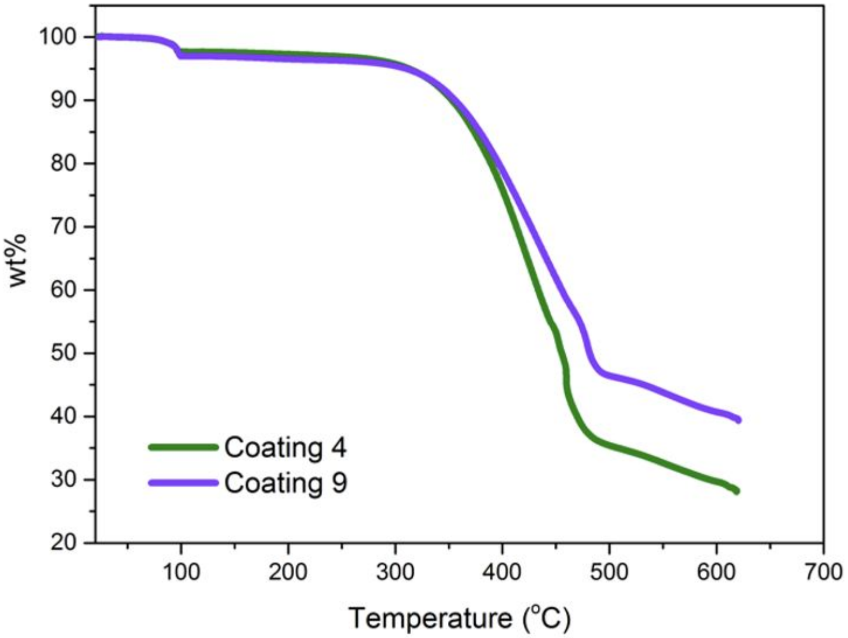

Figure S5. TGA results of isolated NPs attached with both ligand PFDTES and PFPE (co-addition) by using biphasic approach. The decrease of PFDTES amount (by half) in the reaction resulted in the less weight loss (wt%), as shown by the TGA of Coating 4.

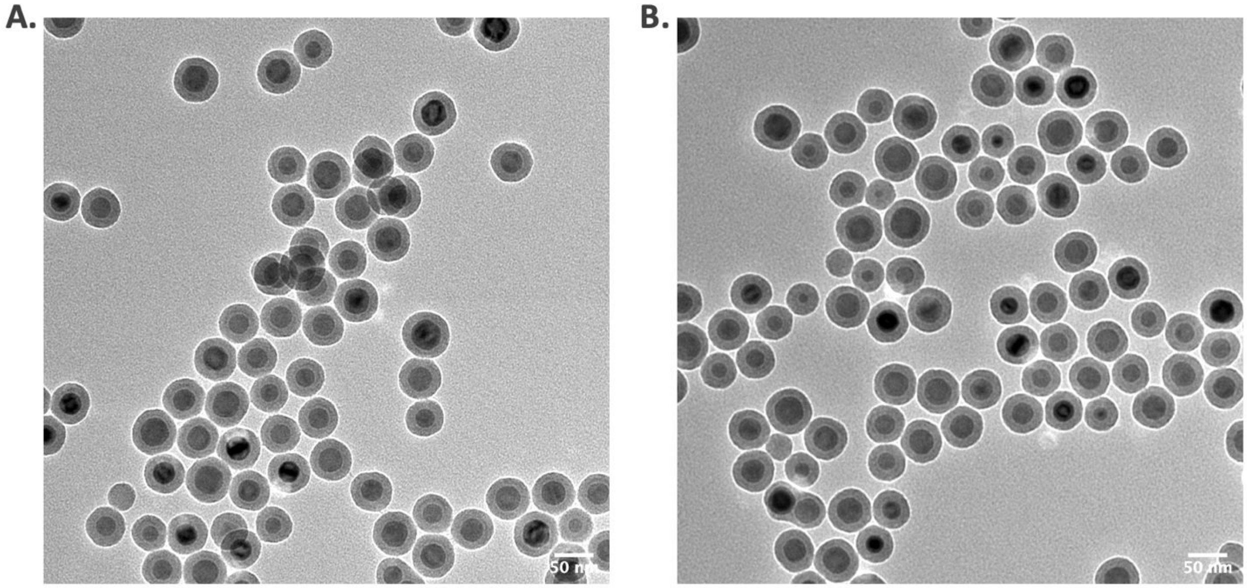

Figure S6. TEM image of (A) unfunctionalized Fe_3O_4@SiO_2 NPs and (B) fluorous ligand-functionalized Fe_3O_4@SiO_2 NPs (SPION@SiO_2-**4**) via biphasic approach.

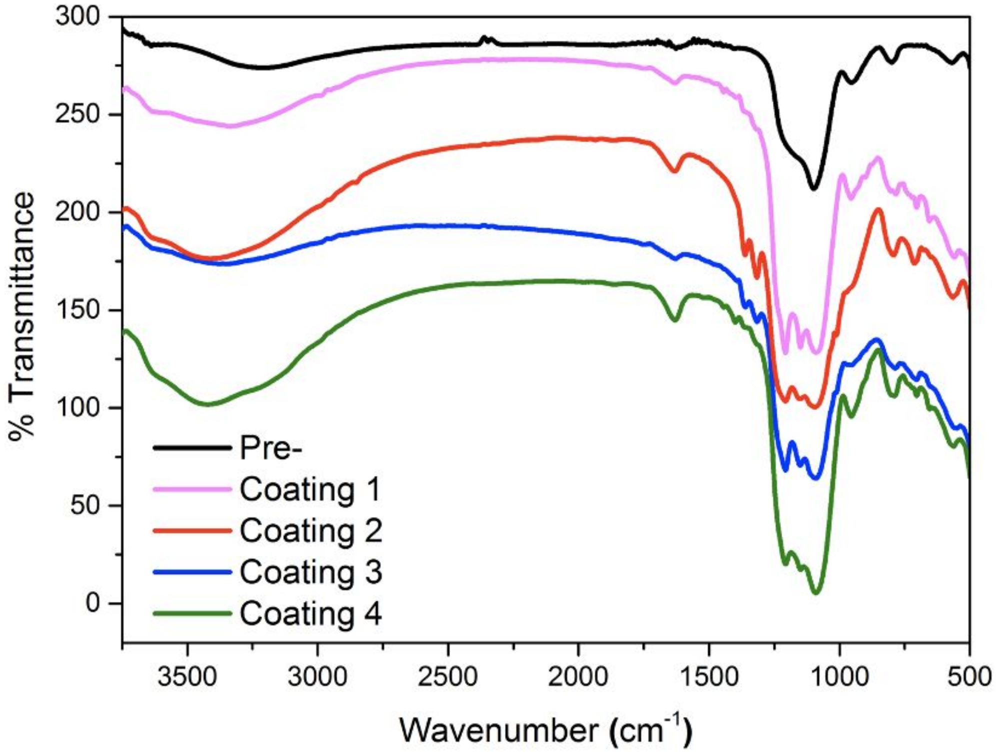

Figure S7. FTIR results of $Fe_3O_4@SiO_2$ NPs with and without the fluorous coating. The spectra have been shifted vertically for better visibility.

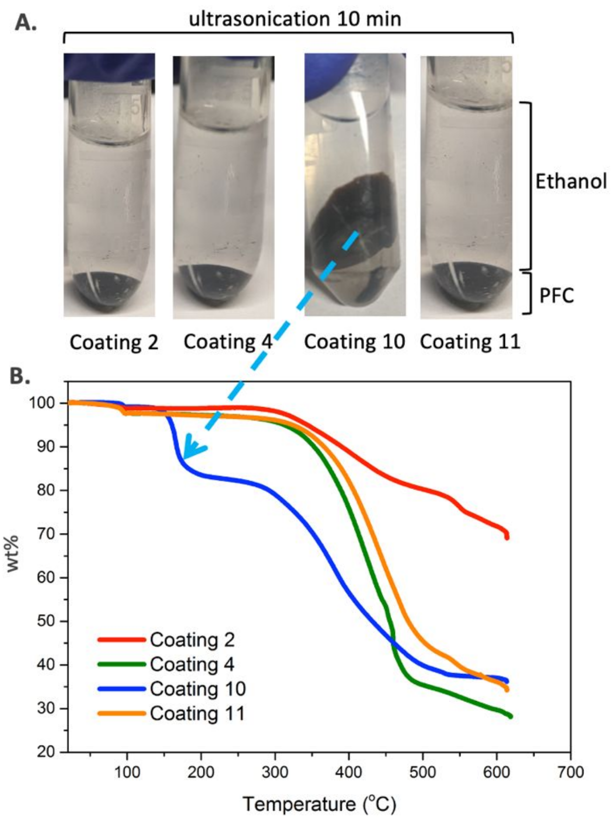

Figure S8 (A) Comparison of Fe_3O_4@SiO_2 NPs with Coating **2**, **4**, **10**, and **11**. Fe_3O_4@SiO_2 NPs-10 in PFC phase went back to ethanol phase after 10 min of ultrasonication, indicating the loosely attached ligands. Fe_3O_4@SiO_2 NPs-**11** remained in PFC phase after 10 min sonication, suggesting the improved stability of Coating **10** on NPs with the assistance of PFDTES. (b) TGA of comparison between NPs with non-chemically bonded/absorbed ligands (negative control: Coating **10**) and with chemically bonded ligands (Coating **2**, **4**, and **11**), where the unstable ligand layer generates a degradation peak at a temperature at 150°C.

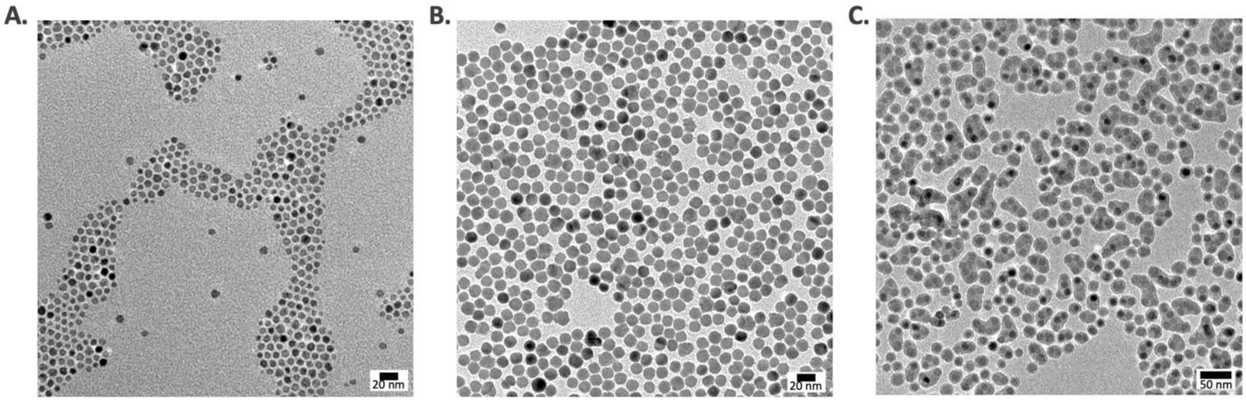

Figure S9. TEM images of (A) 8.4 nm $MnFe_2O_4$ NPs (the seeds), (B) 15.4 nm $MnFe_2O_4$ NPs, and (C) $MnFe_2O_4$@SiO_2 NPs. \

References:

(1) Borduz, L.; Tsuda, S.; Hirota, Y. Ferrofluid Composition and Process. *United States Pat. US 6,277,298 B1* **2001**.
